# Pre-Operative Group and Save in Elective and Emergency Laparoscopic Cholecystectomy: Necessity, Cost-Effectiveness, and Own Experience

**DOI:** 10.3390/jcm13102749

**Published:** 2024-05-07

**Authors:** Mohammed Hamid, Marie Kershaw, Resya Bhakthavalsalan, Rishika Shivamurthy, Sian Davies, Rishi Singhal, Rajwinder Nijjar, Tom Wiggins, Ricardo Camprodon, Zuhair Ahmed

**Affiliations:** 1 University Hospitals Birmingham NHS Foundation Trust, Mindelsohn Way, Birmingham B15 2TH, UK; 2 Dudley Group NHS Foundation Trust, Russell’s Hall Hospital, Pensnett Rd., Dudley DY1 2HQ, UK

**Keywords:** laparoscopic cholecystectomy, group and save, blood transfusion, O-negative blood, patient outcomes, cost-effectiveness, cross-matched blood

## Abstract

**Background**: Laparoscopic cholecystectomy is associated with a high safety profile. This study seeks to quantify the incidence of blood transfusion in both the elective and emergency settings, examine related patient outcomes, and investigate selection criteria for pre-operative Group and Save (G&S) sampling. **Methods:** A prospective multi-centre observational study was conducted to investigate patients undergoing either elective or emergency laparoscopic cholecystectomy in the UK between January 2020 and May 2021. Multivariate logistical regression models were used to identify patient factors associated with the risk of transfusion and explore outcomes linked to pre-operative G&S sampling. **Results:** This study comprised 959 patients, with 631 (65.8%) undergoing elective cholecystectomy and 328 (34.2%) undergoing emergency surgery. The median age was 48 years (range: 35–59), with 724 (75.5%) of the patients being female. Only five patients (0.5%) required blood transfusions, receiving an average of three units, with the first unit administered approximately six hours post-operatively. Among these cases, three patients (60%) had underlying haematological conditions. In adjusted models, male gender was significantly associated with the need for a blood transfusion (OR 11.31, *p* = 0.013), while the presence of a pre-operative Group and Save sample did not demonstrate any positive impact on patient outcomes. **Conclusions:** The incidence of blood transfusion following laparoscopic cholecystectomy is very low. Male gender and haematological conditions may present as independent risk factors. Pre-operative G&S sampling did not yield any positive impact on patient outcomes and could be safely excluded in both elective and emergency cases, although certain population subsets will warrant further consideration.

## 1. Introduction

Laparoscopic cholecystectomy is a prevalent procedure, with approximately 60,000 cases conducted annually in the United Kingdom (UK) [1]. This operation is renowned for its high safety profile and favourable patient satisfaction [2,3]. The procedure is predominantly performed in the elective day case setting, with patients going home on the same day. In the emergency setting, a “hot gallbladder” operation is often performed to stabilise an acutely unwell patient, with satisfactory and comparable outcomes to delayed elective surgery. The indications for a laparoscopic cholecystectomy are primarily linked to the symptoms and complications of gallstones, such as recurrent episodes of biliary colic, acute calculous cholecystitis, pancreatitis, and cholangitis secondary to choledocholithiasis. Other indications for this operation include biliary dyskinesia, acalculous cholecystitis, gallbladder polyps, or localised cancer [4]. The risks and complications associated with the procedure comprise post-operative pain, minor or major haemorrhage (vascular injury), infection of the wounds or internal collection, scars (port-site, Kocher, or midline), incisional hernia, short- or long-term diarrhoea, bile reflux, injury to surrounding structures (stomach, duodenum, colon, vessels, and bile duct), bile leak, and complications associated with general anaesthesia [4].

Major vascular injuries have become increasingly rare (0.08%), with up to 2.9% of patients necessitating blood components or packed red blood cell (pRBC) transfusions [5,6]. Several authors have classified major vascular injuries during laparoscopic cholecystectomy, and these have been outlined in Table 1 [5,7,8,9].

There are four major categories encompassing the risk factors associated with vascular injury or bleeding during laparoscopic cholecystectomy: Vascular anatomical variations, particularly of the cystic artery and right hepatic artery, which commonly arise in at least 50% of individuals and can only be recognised by careful dissection; the intrinsic gallbladder pathology; patient-related factors; and surgeon-related factors [10,11,12,13,14,15,16]. The aforementioned categories and linked causes have been listed in Table 2.

Despite the infrequent occurrence of significant blood loss requiring transfusion, it is standard practice in the UK to routinely request pre-operative Group and Save (G&S) samples in anticipation of potential major haemorrhage [17]. A Group and Save sample serves to determine the patient’s blood group (ABO and RhD) and identify any unusual red blood cell antibodies that might trigger a haemolytic transfusion reaction. Other than the commonly acknowledged ABO and RhD blood group systems, there are 33 others that are recognised by the International Society of Blood Transfusion. The blood group systems commonly tested for compatibility include Duffy, Kel, Kidd, Lewis, P1P(K), GLOB, I antigens, MNS, Chido-Rodger, Colton, Diego, Er, Gerbich, Lutheran, Vel, Cartwright (Yt), and Knops blood group systems [18]. Detecting such atypical antibodies is crucial, as it could potentially delay the procurement of cross-matched blood products if they are not readily accessible locally and must be sourced from the National Blood Service. Therefore, obtaining a pre-operative Group and Save sample, in theory, should significantly save valuable time [19]. Blood banks mandate a minimum of two valid samples before surgery to ensure that matched units are promptly available in emergency situations [17].

The main objectives of this study were to assess the need for packed red blood cell (pRBC) transfusions in patients undergoing laparoscopic cholecystectomy (both elective and emergency), investigate the potential for selective utilisation of Group and Save sampling, and examine whether the quantity of pre-operative Group and Save samples influenced patient outcomes.

## 2. Methods

A prospective observational study was conducted to analyse all patients who underwent a laparoscopic cholecystectomy between 1 January 2020 and 31 May 2021. This investigation took place during the COVID-19 pandemic at three centres located within the West Midlands, UK. Throughout the study period, one of the centres implemented the practice of obtaining two pre-operative Group and Save samples prior to emergency cases, while another centre opted for not performing any Group and Save sampling. Elective patients were typically scheduled to have one sample taken during their pre-operative assessment and another on the day of the procedure, although the logistics of this varied among the centres. This ‘pseudo-randomisation’ approach provided an opportunity to compare clinical outcomes between patients with and without the optimal two Group and Save samples. Institutional approval was obtained at the outset of the study, confirming that no ethical approval or informed consent was required since patient care and pathways remained unchanged. The study adhered to the ethical standards outlined in the Helsinki Declaration.

Electronic and physical records from the operating theatres were reviewed for 1000 consecutive patients who underwent gallbladder removal, starting from 1 January 2020. Exclusion criteria encompassed patients under the age of 18, procedures not completed laparoscopically, or instances where the primary indication for surgery differed from cholecystectomy, such as bowel cancer resection. A total of 959 patients met the eligibility criteria, which were verified by a minimum of two study investigators.

Data were extracted from both electronic and physical patient records for all eligible individuals and stored on an encrypted, password-protected computer. The extracted data comprised demographics (age, sex, American Society of Anaesthesiologists (ASA) grade, and initial presentation details such as indication for surgery and additional investigations undertaken (MRCP: magnetic resonance cholangiopancreatography and ERCP: endoscopic retrograde cholangiopancreatography)), the number of pre-operative Group and Save (G&S) samples, admission status (emergency or elective), operative details, such as duration of surgery and drain insertion, and hospital admission statistics, including the number of packed red blood cell (pRBC) units transfused, post-operative complications categorised by the Clavien–Dindo grade over a 30-day period, length of hospital stay (LOS), and 30-day mortality.

The data were summarised using the median and interquartile range (IQR) for continuous variables and the number and percentage for categorical data. Variables were compared between the elective and emergency groups using Mann–Whitney U tests for continuous variables and Chi-squared analysis for categorical variables. Odds ratios (ORs) and 95% confidence intervals (95% CIs) were calculated to assess patient factors associated with the requirement of a blood transfusion and post-operative outcomes following two pre-operative Group and Save samples, utilising both univariate and multivariate binomial logistic regression. *p*-values less than 0.05 were considered statistically significant. GraphPad Prism V9.1.3 (GraphPad Software, LLC., Boston, MA, USA) and R 4.1.0 (R Foundation for Statistical Computing, Vienna, Austria) were employed for statistical analysis.

## 3. Results

A total of 959 patients were included in the study, with 631 (65.8%) undergoing elective cholecystectomy and 328 (34.2%) undergoing emergency surgery (Table 3). The median age was 48 years (range: 35–59), and 724 (75.5%) patients were female. All cases were performed for symptoms and complications of gallstone disease. The majority (63.4%) of procedures were indicated for biliary colic, followed by cholecystitis (32.3%), pancreatitis (4.0%), and then cholangitis (0.3%) secondary to choledocholithiasis. For elective surgery, the primary indication was biliary colic (75.1% vs. 40.9%, *p* < 0.001), and patients were more likely to have only one pre-operative Group and Save sample (40.9% vs. 14.3%, *p* < 0.001). Conversely, for emergency surgery, the most common indication was acute cholecystitis (54.9% vs. 20.6%, *p* < 0.001), and patients either had no pre-operative Group and Save samples (38.7% vs. 15.2%, *p* < 0.001) or too many samples sent (11.6% vs. 6.8%, *p* = 0.012).

Patients undergoing emergency surgery exhibited more comorbidities (ASA 3, 10.4% vs. 5.4%, *p* = 0.005) and necessitated additional pre-operative investigations (magnetic resonance cholangiopancreatography, 42.1% vs. 31.1%, *p* < 0.001, and ERCP endoscopic retrograde cholangiopancreatography, 14.3% vs. 9.5%, *p* = 0.025). Emergency procedures were of longer duration (74 vs. 67 min, *p* = 0.003), and patients were more likely to require drain insertion (11.6% vs. 3.0%, *p* < 0.001). Furthermore, patients undergoing emergency surgery were at a higher risk of experiencing post-operative complications (Clavien–Dindo 3, 4.9% vs. 1.9%, *p* = 0.014) and had longer hospital stays following the procedure (range, 0–13 vs. 0–7 days, *p* < 0.001). However, there was no significant difference in the incidence of post-operative pRBC transfusions (0.6% vs. 0.5%, *p* > 0.999). There were no recorded incidents of major vessel injury in any of the cases. No patient deaths were recorded at 30 days post-operation for either elective or emergency surgery.

## 4. Transfusion Patient Characteristics

Five patients (0.5%) required post-operative pRBC transfusions (mean: 3 units, range 1–4 units), comprising three elective and two emergency cases (Table 4). Three of these operations were indicated for recurrent biliary colic, one for cholecystitis, and one other for pancreatitis. Four of these patients were male, with an age range of 18–51 years, an ASA range of two to four, and three had underlying haematological conditions. Three out of these five procedures were completed in less than one hour “skin to skin” (range: 50–119 min). The indications for transfusion and return to the operating theatre included haemodynamic instability, presumed bleeding due to operative difficulty, an increased rate of drain content associated with a drop in haemoglobin on arterial blood gas analysis, and significant post-operative pain. One patient had the “optimal” two Group and Save samples pre-operatively, while two other patients had one, and another two patients had no samples before their operation. There was over 100 min in time difference between the shortest and longest laboratory delay, from crossmatch request to administration of the first pRBC unit (range: 13–115 min). Despite one patient having two valid pre-operative Group and Save samples, this did not expedite the time to transfusion due to delays in requesting pRBC units, laboratory logistics, and administrative processes. All patients necessitated an additional post-operative Group and Save sample before receiving their cross-matched units. The first transfusion was administered to all patients with a mean time of six hours post-operatively (range: 204–764 min), even though one patient experienced a one-litre estimated blood loss intra-operatively. Four patients returned to the operating theatre for diagnostic laparoscopy and washout (mean time: 47 min), with three out of four bleeds originating from the liver edge (halted by diathermy) and one from a vessel at an umbilical port site (stopped by one transfixion suture).

## 5. Factors Associated with Transfusion

Patient factors associated with an increased risk of blood transfusion on both univariate and multivariate analyses, respectively, included male sex (OR 12.52 (95% CI 1.39, 112.57), *p* = 0.031, OR 11.31 (95% CI 1.24, 102.87), *p* = 0.013), and haematological conditions (OR 3.28 (95% CI 0.93, 5.46), *p* = 0.037, OR 2.83 (95% CI 0.88, 4.62), *p* = 0.048) (Table 5). Within this study, the following factors did not show a significant increase in risk for blood transfusion on both univariate and multivariate analyses, respectively: Delay to surgery from initial admission (OR 1.00 (95% CI 0.99, 1.00), *p* = 0.378, OR 1.00 (95% CI 0.99, 1.00), *p* = 0.276), emergency “hot gallbladder” surgery (OR 1.28 (95% CI 0.21, 7.72), *p* = 0.677, OR 0.64 (95% CI 0.08, 5.34), *p* = 0.676), or prolonged duration of surgery (OR 1.00 (95% CI 0.97, 1.03), *p* = 0.788, OR 1.00 (95% CI 0.98, 1.03), *p* = 0.782).

## 6. Group and Save and Patient Outcomes

When outcomes were analysed using univariate logistic regression and linear regression for categorical and continuous variables, respectively, having a minimum of two pre-operative Group and Save samples did not result in positive changes in any of the post-operative outcomes assessed: transfusion (OR 0.30 (95% CI 0.03, 2.27), *p* = 0.191), Clavien–Dindo grade 3 and above (OR 1.23 (05% CI 0.60, 2.55), *p* = 0.751), and length of hospital stay (OR 1.07 (95% CI 0.99, 1.16), *p* = 0.086). This finding persisted in adjusted models accounting for initial presentation, waiting time to surgery, type of admission (emergency vs. elective), and ASA grade (Table 6): transfusion (OR 0.22 (95% CI 0.02, 2.15), *p* = 0.148), Clavien–Dindo grade 3 and above (OR 1.14 (05% CI 0.50, 2.61), *p* = 0.751), and length of hospital stay (OR 1.08 (95% CI 0.99, 1.17), *p* = 0.055).

## 7. Discussion

This study highlights the exceptionally low occurrence of significant blood loss (0.5%) and major vascular injury (0%) during laparoscopic cholecystectomy, both in the elective and emergency settings. The primary findings indicate that pre-operative Group and Save sampling did not yield any positive impact on patient outcomes. Additionally, patients with haematological conditions and male sex are at a heightened risk for blood loss necessitating transfusion.

The current evidence aligns with the low incidence of clinically significant blood loss observed in our study (0.5%). Level one evidence from over 40,000 laparoscopic cholecystectomies has reported post-operative haemorrhage ranging between 0.004% and 2.3% [8,20]. Although some studies have documented a higher rate of post-operative bleeding, such as 3.2% (*n* = 43,028) and 4.1% (*n* = 4243), major vascular injury during laparoscopic cholecystectomy remains infrequent (0.08%) [5,21]. In 2005, a meta-analysis by Larobina et al. analysed 760,890 closed-entry laparoscopies and 22,465 open-entry laparoscopies and recorded the incidence of major vascular injury to be 0.044% and 0%, respectively [22].

The 2016 National Institute for Health and Care Excellence (NICE) guidelines, which aimed to standardise the process of pre-operative investigation across the United Kingdom (UK), emphasised the reduction of unnecessary pre-operative testing [23]. Several UK publications have similarly concluded that routine Group and Save sampling for laparoscopic cholecystectomy is “unnecessary” [19,24,25,26]. The 2012 French Society of Anaesthesiology and Intensive Care (SFAR) guidelines, which were endorsed by 17 surgical and medical scientific societies, also highlighted the need to reduce redundant pre-operative tests, advising against the routine use of Group and Save sampling in low-risk patients [27]. Although the 2012 SFAR guidelines were not designated for laparoscopic cholecystectomy, the authors of SFAR subsequently published data in 2017 and specified laparoscopic cholecystectomy as a procedure that does not require routine pre-operative Group and Save sampling [28]. Other international studies also support the safe omission of Group and Save in various laparoscopic procedures [29,30,31,32].

Despite the available literature in favour of omitting routine pre-operative Group and Save sampling for laparoscopic cholecystectomy, specific national and international guidelines on this exact topic are limited. Like the 2012 SFAR and 2016 NICE guidelines, the British Association of Day Surgery (BADS) and the Association of Anaesthetists of Great Britain and Ireland (AAGBI) document on day-case surgery does not precisely outline the role of pre-operative Group and Save sampling in laparoscopic cholecystectomy [33]. Although specific recommendations are excluded, these guidelines actively encourage clinicians to reach a patient-centred decision based on operative severity and the likelihood of blood loss, given that certain patient groups are at high risk of bleeding.

In our study, we did not observe a linear relationship between the number of pre-operative Group and Save samples sent and the receipt time of cross-matched blood. One patient with no Group and Save samples received their first cross-matched pRBC unit before another patient who had the “optimal” two pre-operative samples sent. While there are various reasons to account for this difference, such as sample haemolysis or unsuitable specimen volume, duplicate samples, patient haemodynamic status, and human errors in requesting crossmatch pRBC units (incomplete or incorrect details, not handwritten), delays still occur despite the use of two pre-operative samples. Our local blood bank, upon activation of a major haemorrhage protocol, can provide cross-matched blood within 45–60 min. In contrast, it can provide O-negative blood, platelets, and fresh frozen plasma within 15–20 min.

In most medical and surgical emergencies, O RhD negative (O-negative) blood can safely be given to anyone when the blood type is not immediately known. About 85% of the UK population is RhD positive (35% of the population has O+, the most common type). It is safe for most recipients because it does not have any A, B, or RhD antigens on the surface of the cells and is compatible with every other ABO and RhD blood group [34]. Our theatres also maintain a supply of O-negative units on standby for immediate use in emergencies. Considering that the mean time to transfusion was six hours post-operatively for our patients experiencing blood loss, utilising the immediately available O-negative blood as a first option is a safe choice while a post-operative Group and Save and crossmatch request is sent, and this suggestion has also been made by other authors [19,32].

To ensure that blood banks can provide cross-matched products, the validity of Group and Save samples typically lasts between five and seven days. Data analysis of our elective patient cohort revealed that 408 (64.7%) patients had invalid samples due to changes in their operation dates, cancellations, and misplaced pre-operative clinic records. While a historical sample can be advantageous for investigating atypical antibodies, duplicated and untimely samples can lead to significant costs. Within our trust, the cost of a single Group and Save sample, including additional logistical fees, totals up to GBP 20. Locally, the overall annual cost of Group and Save samples for laparoscopic cholecystectomies is GBP 18,056, with GBP 7946 being spent on invalid Group and Save testing electively. Nationally, if two Group and Save samples were to be sent for each cholecystectomy performed annually in the UK, it would amount to almost two and a half million pounds. Barret-Lee et al. and Hamza et al. have documented similar estimated costs [19,32].

Considering the absence of a positive impact on patient outcomes and the cost implications, while a viable alternative option is available (O-negative blood and post-operative crossmatch request), several recommendations have been made for a more selective and individualised approach to pre-operative testing in laparoscopic surgery [35,36]. Within our study, we identified male patients and those with haematological conditions as groups that should have a lower threshold for Group and Save testing. Ongoing efforts to raise awareness, both in the surgical and anaesthetic fields, have been cited as a fundamental method to reduce the rate of unnecessary pre-operative investigations, as the perception of an increased risk of major haemorrhage during laparoscopic surgery persists among anaesthetic and surgical staff [19,37].

## 8. Limitations

We did not delve into the finer details behind male sex being an independent factor for requiring a blood transfusion; however, numerous studies have concluded that male sex is an independent factor for difficulty and complications in laparoscopic cholecystectomy [38,39,40]. This study does not ascertain the clinical decision-making behind patients who had one or more Group and Save samples. Reasons other than invalid timing for multiple sample requests include incomplete or incorrectly labelled samples, under-filled bottles, haemolysed samples, and over-sampling by junior staff [19]. Further studies may wish to randomise patients across sex, predisposing conditions, and a range of pre-operative Group and Save samples and consider surveying surgeons and anaesthetists about their reasons behind their pre-operative testing practices, particularly Group and Save for specific procedures.

## 9. Conclusions

The incidence of major vascular injury and blood loss necessitating transfusion in laparoscopic surgery is exceedingly low. Pre-operative Group and Save sampling demonstrated no positive impact on patient outcomes and could safely be omitted in both elective and emergency cases. Male sex and haematological conditions may emerge as independent risk factors for blood loss in laparoscopic cholecystectomy. We recommend the utilisation of readily available O-negative blood in emergency situations, while Group and Save and crossmatch requests can be sent post-operatively in case additional units are required. In accordance with NICE and other similar guidelines, we advocate for the reduction of inappropriate pre-operative tests and suggest personalising the approach to pre-operative Group and Save in patients anticipated to have operative difficulty and in those with high-risk factors for blood loss.

## Figures and Tables

**Table 1:** Classifications of major vascular injury during laparoscopic cholecystectomy defined by a variety of authors.

Study	Definition
Pesce et al., 2023 [7]	Any bleeding involving the right hepatic artery, portal vein, supra-hepatic veins, inferior vena cava that always requires conversion to open surgery for control or repair; need for blood transfusions; associated biliary injury; or need for transfer to tertiary centre.
Kaushik et al., 2010 [8]	Any bleeding involving the cystic artery, right hepatic artery, portal vein, superior mesenteric vein, supra-hepatic veins, inferior vena cava, or aorta that requires conversion to open surgery to control or repair; the requirement of additional surgical procedures; or the need for blood transfusions.
Bektas et al., 2007 [9]	Vascular involvement with concomitant biliary injury of different grades (types C and D). Type C: Tangential injury of the common bile duct with or without vascular lesion; Type D: Complete transection of the common bile duct with or without vascular lesion.
Schäfer et al., 2000 [5]	Injury to any of the following vessels: aorta, vena cava, portal vein, hepatic artery, and splenic artery; iliac vessels; mesenteric, omental, and renal vessels. Intra-operative vascular injury: local haemorrhage within the abdominal cavity, retroperitoneum, or abdominal wall. Post-operative: bleeding occurring within 24 h after surgery.

**Table 2:** Causes and risk factors associated with vascular injury or bleeding during laparoscopic cholecystectomy.

Category	Causes and Risk Factors
Common cystic artery and right hepatic artery anatomical variations [10,11,12,13]	Two cystic arteries (superficial and deep; anterior and posterior; double anterior; accessory) Short single cystic artery originating from a “Caterpillar” right hepatic artery Long single cystic artery originating from elsewhere, other than the right hepatic artery (e.g., cystic artery arising from the gastroduodenal artery, passing outside the Calot’s triangle) Cystic artery visualised anteriorly rather than posteriorly in relation to Mascagni’s lymph node Vessels found on the postero-lateral margin of gallbladder bed
Gallbladder pathology [14]	Acute or chronic cholecystitis Significant previous pancreatitis episode Gallbladder anomalies (gallbladder duplication; gallbladder agenesia; left-sided gallbladder)
Patient-related factors [15]	Previous surgery Previous biliary endoscopic procedures (e.g., endoscopic retrograde cholangiopancreatography) Underlying liver disease Obesity (BMI > 35)
Surgery- and surgeon-related factors [16]	Inadequate exposure Failure to recognise anatomical landmarks Learning curve Subspecialty interest (general surgeon vs. colorectal surgeon vs. upper gastrointestinal surgeon vs. hepatobiliary surgeon)

**Table 3:** Study patient characteristics for all patients, comparing elective and emergency cases.

Characteristic	All (*n* = 959)	Elective (*n* = 631)	Emergency (*n* = 328)	*p*-Value
Age, median (IQR) years	48 (35–59)	47 (35–59)	50 (36–60)	0.349
Female sex, *n* (%)	724 (75.5)	481 (76.2)	243 (74.1)	0.464
First presentation, *n* (%)				
Biliary colic	608 (63.4)	474 (75.1)	134 (40.9)	<0.001 *
Cholecystitis	310 (32.3)	130 (20.6)	180 (54.9)	<0.001 *
Pancreatitis	38 (4.0)	26 (4.1)	12 (3.7)	0.728
Cholangitis	3 (0.3)	1 (0.2)	2 (0.6)	0.235
Additional imaging, *n* (%)				
MRCP	334 (34.8)	196 (31.1)	138 (42.1)	<0.001 *
ERCP	107 (11.2)	60 (9.5)	47 (14.3)	0.025 *
ASA grade, *n* (%)				
1	174 (18.1)	118 (18.7)	56 (17.1)	0.596
2	716 (74.7)	478 (75.8)	238 (72.6)	0.309
3	68 (7.1)	34 (5.4)	34 (10.4)	0.005 *
4	1 (0.1)	1 (0.2)	0 (0.0)	>0.999
G&S pre-operatively				
0	223 (23.3)	96 (15.2)	127 (38.7)	<0.001 *
1	305 (31.8)	258 (40.9)	47 (14.3)	<0.001 *
2	350 (36.5)	234 (37.1)	116 (35.4)	0.621
3+	81 (8.4)	43 (6.8)	38 (11.6)	0.012 *
Duration of surgery (m)	69 (51–90)	67 (51–85)	74 (52–98)	0.003 †
Major vessel injury (%)	0 (0.0)	0 (0.0)	0 (0.0)	>0.999
Drain	57 (5.9)	19 (3.0)	38 (11.6)	<0.001 *
Blood transfusion, *n* (%)	5 (0.5)	3 (0.5)	2 (0.6)	>0.999
Outcomes (30-day)				
Clavien–Dindo grade				
3	28 (2.9)	12 (1.9)	16 (4.9)	0.014 *
4	2 (0.2)	1 (0.2)	1 (0.3)	>0.999
LOS, median (IQR) d	1 (0–1)	0 (0–1)	1 (1–2)	<0.001 †
Mortality	0 (0.0)	0 (0.0)	0 (0.0)	>0.999

* Indicates statistically significant using Chi-squared analysis; † Indicates statistically significant using the Mann–Whitney *U* test. IQR: interquartile range; MRCP: magnetic resonance cholangiopancreatography; ERCP: endoscopic retrograde cholangiopancreatography; ASA: American Society of Anaesthesiologists; G&S: Group and Save sample; LOS: length of hospital stay; d: days; m: minutes.

**Table 4:** Characteristics of patients requiring blood transfusion(s).

Characteristics	Patients				
	1	2	3	4	5
Age (years)	36	19	18	50	51
Sex	F	M	M	M	M
Admission	Elective	Emergency	Elective	Emergency	Elective
Initial presentation	Cholecystitis	Biliary colic	Biliary colic	Biliary colic	Pancreatitis
G&S samples pre-op	0	1	1	2	0
ASA grade	2	2	4	2	2
Surgery duration (m)	119	89	52	50	50
Time to first unit (m)	368	247	204	764	235
Laboratory delay (m)	37	19	13	47	115
Further G&S sample	Yes	Yes	Yes	Yes	Yes
pRBC transfused (u)	1	4	2	4	4
Re-operation	No	Laparoscopic ×1	Laparoscopic ×1	Laparoscopic ×1	Laparoscopic ×1
Site of bleed	Cystic artery (Intra-op)	Gallbladder bed	Gallbladder bed	Gallbladder bed	Port-site vessel
EBL (mL)	1000	700	300	500	500
Any haematological conditions	-	Hereditary Spherocytosis	-	Carrier of α-thalassaemia	Gilbert’s syndrome
Clavien–Dindo grade	2	4	3	3	3
LOS (d)	3	8	5	2	2

LOS: length of hospital stay; d: days; m: minutes; ASA: American Society of Anaesthesiologists; G&S: Group and Save sample; pRBC: packed red blood cells; u: units; Pre-op: pre-operatively; Intra-op: intraoperatively; EBL: estimated blood loss; ml: millilitres; α: alpha.

**Table 5:** Factors associated with requiring a transfusion using univariate and multivariate binomial logistic regression modelling.

Characteristic	Univariate OR (95% CI)	*p*-Value	Multivariate OR (95% CI)	*p*-Value
Male sex	12.52 (1.39, 112.57)	0.031 *	11.31 (1.24, 102.87)	0.013 *
Haematological Dx	3.28 (0.93, 5.46)	0.037 *	2.83 (0.88, 4.62)	0.048 *
Emergency surgery	1.28 (0.21, 7.72)	0.677	0.64 (0.08, 5.34)	0.676
Delay to surgery	1.00 (0.99, 1.00)	0.378	1.00 (0.99, 1.00)	0.276
Surgery duration	1.00 (0.97, 1.03)	0.788	1.00 (0.98, 1.03)	0.782

* Indicates statistically significant; 95% CI: 95% confidence interval; Dx: diagnosis.

**Table 6:** Outcomes associated with having a minimum of two Group and Save samples pre-operatively using univariate and multivariate binomial logistic regression models.

Characteristic	Univariate OR (95% CI)	*p*-Value	Multivariate OR (95% CI)	*p*-Value
Transfusion	0.30 (0.03, 2.27)	0.191	0.22 (0.02, 2.15)	0.148
Clavien–Dindo 3+	1.23 (0.60, 2.55)	0.751	1.14 (0.50, 2.61)	0.751
Length of stay (d)	1.07 (0.99, 1.16)	0.086	1.08 (0.99, 1.17)	0.055

95% CI: 95% confidence interval; d: days.

## Data Availability

The data presented in this study are available on request from the corresponding author. The data are not publicly available due to ongoing audits and service improvement.
